# Do authors of research funded by the Canadian Institutes of Health Research comply with its open access mandate?: A meta-epidemiologic study

**DOI:** 10.1371/journal.pone.0256577

**Published:** 2021-08-24

**Authors:** Michael A. Scaffidi, Karam Elsolh, Juana Li, Yash Verma, Rishi Bansal, Nikko Gimpaya, Vincent Larivière, Rishad Khan, Samir C. Grover

**Affiliations:** 1 Faculty of Health Sciences, School of Medicine, Queen’s University, Kingston, Canada; 2 Division of Gastroenterology, St. Michael’s Hospital, University of Toronto, Toronto, Canada; 3 Observatory of Science and Technology, Montreal, Canada; 4 Li Ka Shing Knowledge Institute, Toronto, Canada; University of Rennes 1, FRANCE

## Abstract

**Background:**

Since 2008, the Canadian Institutes of Health Research (CIHR) has mandated that studies it funds either in whole or in part are required to publish their results as open access (OA) within 12 months of publication using either online repositories and/or OA journals. Yet, there is evidence that authors are poorly compliant with this mandate. Specifically, there has been an apparent decrease in OA publication after 2015, which coincides with a change in the OA policy during the same year. One particular policy change that may have contributed to this decline was lifting the requirement that authors deposit their article in an OA repository immediately upon publication. We investigated the proportion of OA compliance of CIHR-funded studies in the period before and after the policy change of 2015 with manual confirmation of both CIHR funding and OA status.

**Methods and findings:**

We identified CIHR-funded studies published between the years 2014 to 2017 using a comprehensive search in the Web of Science (WoS). We took a stratified random sample from all four years (i.e. 2014 to 2017), with 250 studies from each year. Two authors independently reviewed the final full-text publications retrieved from the journal web page to determine to confirm CIHR funding, as indicated in the acknowledgements or elsewhere in the paper. For each study, we also collected bibliometric data that included citation count and Altmetric attention score Statistical analyses were conducted using two-tailed Fisher’s exact test with relative risk (RR). Among the 851 receiving CIHR funding published from 2014 to 2017, the percentage of CIHR-funded studies published as OA significantly decreased from 79.6% in 2014 to 70.3% in 2017 (RR = 0.88, 95% CI: 0.79–0.99, P = 0.028). When considering all four years, there was no significant difference in the percentage of CIHR-funded studies published as OA in both 2014 and 2015 compared to both 2016 and 2017 (RR = 0.97, 95% CI: 0.90–1.05, P = 0.493). Additionally, OA publications had significantly higher citation count (both in year of publication and in total) and higher attention scores (P<0.05).

**Conclusions:**

Overall, we found that there was a significant decrease in the proportion of CIHR funded studies published as OA from 2014 compared to 2017, though this difference did not persist when comparing both 2014–2015 to 2016–2017. The primary limitation was the reliance of self-reported data from authors on CIHR funding status. We posit that this decrease may be attributable to CIHR’s OA policy change in 2015. Further exploration is warranted to both validate these studies using a larger dataset and, if valid, investigate the effects of potential interventions to improve the OA compliance, such as use of a CIHR publication database, and reinstatement of a policy for authors to immediately submit their findings to OA repositories upon publication.

## Introduction

In Canada, the major federal agency that provides funding for health and medical research is the Canadian Institutes of Health Research (CIHR) [1]. Since 2008, CIHR has mandated that studies it funds either in whole or in part are required to publish their results as OA within 12 months of publication using either digital archiving in online repositories and/or open-access journals [2]. This type of mandate, which is common practice among public funding agencies, ensures that research funded with taxpayer dollars functions as a return on investment to the public [3].

There is evidence, however, that CIHR OA mandate has poor compliance. In October 2018, a study by Larivière et al examined OA publication among a number of publicly funded agencies worldwide and found that only 60% of CIHR funded studies were published OA in 2014 [4]. This percentage further dropped to 40% in 2017, with the authors attributing this decline to dissemination embargoes, as well as policy changes in CIHR OA mandate made in 2015. This change, which aimed to harmonize the OA policies of CIHR with the two other primary Canadian research councils, SSHRC (Social Sciences and Humanities Research Council) and NSERC (Natural Sciences and Engineering Research Council), notably relaxed several requirements for authors, including allowing authors to deposit their article in an OA repository within 12 months of publication rather than the previous stipulation of immediate digital archiving [2].

Further investigation is required to corroborate whether there was a significant decline in OA compliance. Given that prior research has relied primarily on automated methods to determine both OA status and presence of CIHR funded studies [4, 5], we believe that manual methods may allow for more accurate estimates. In particular, automated detection of OA status has been demonstrated to have a non-trivial amount of error, as it has demonstrated to miss approximately 23% of OA studies using web searches [5], while automated detection of CIHR funding, though not previously evaluated, may miss important exemptions from the CIHR OA mandate, such as for fellowships and graduate awardees [6].

To address these concerns, we investigated the proportion of OA compliance of CIHR-funded studies in the period before and after the policy change of 2015 with manual confirmation of both CIHR funding and OA status. We hypothesized that there would be a decrease in OA studies after the policy change.

## Methods

**Fig 1:**
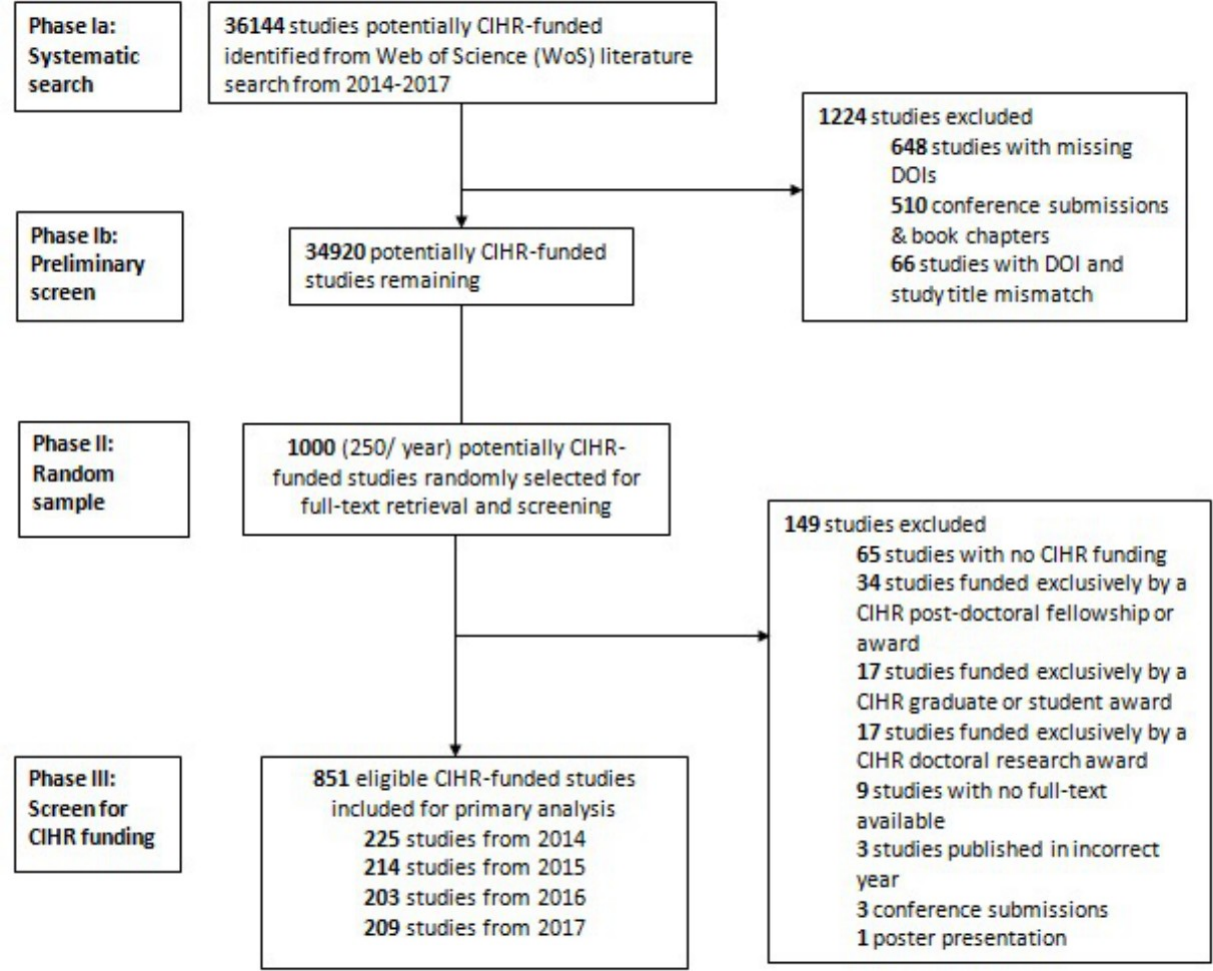
Study flow study characteristics.

We conducted a meta-epidemiologic study of all studies published between the years 2014 to 2017 that received CIHR funding. Reporting of our findings followed guidelines for the reporting of meta-epidemiological methodology research (see **S1 Appendix**) [7]. The protocol of this study was not pre-registered. A summary of the study methodology, including systematic search, is provided in **Fig 1**.

### Definitions

We used the definition of OA adopted elsewhere as “free to read online, either on the publisher website or in an OA repository” [5]. Any studies that did not meet this definition were considered “closed access”. We defined a CIHR-funded study as one in which CIHR funding was either directly or indirectly acknowledged by the authors in the paper. We defined the CIHR open access policy change as the harmonization that occurred in 2015 [2], wherein a summary of the key policy changes before and after 2015 is provided in **Table 1**.

**Table 1:** Summary of open access policy for the Canadian Institutes of Health Research before and after 2015.

	Before 2015	After 2015
**Open access**	Required	Required
**Immediate archiving**	Required	Encouraged
**Digital archiving**	Upon publication	12 months
**Embargo length**	12 months	12 months
**APC funding**	Covered	Covered
**Agencies involved**	CIHR	CIHR, SSHRC, NSERC
**Suggested loci of deposit**	PMC Canada; Institutional repository	Institutional repository; Disciplinary repository

*APC*, article processing charge; *CIHR*, Canadian Institutes of Health Research; *NSERC*, Natural Sciences and Engineering Research Council; *PMC Canada*, PubMed Central Canada;*SSHRC*, Social Sciences and Humanities Research Council.

Note: PMC Canada was taken offline in February 2018 and joined with Europe PubMed Central.

### Systematic search

We identified studies that received funding from CIHR in the Web of Science (WoS) database (Clarivate Analytics). A comprehensive search was created listing “Canadian Institutes of Health Research” and/ or “CIHR” as funding sources. Additionally, the search included a list of individual name grants and initiatives from the CIHR [8] (see **S1 File** for the full search strategy). Searches were executed in the WoS database from 2014 to 2017 in July 2019. A health sciences librarian was consulted to review the search strategy.

### Study identification

Studies with identical digital object identifiers (DOIs) were excluded in order to remove duplicate entries. We also excluded any conference submissions, as these are not covered under the CIHR mandate for OA [1]. Specifically, we removed entries with any of the following terms appearing in the journal field: the words “conference” or “symposium”; any variation on an ordinal signifier (e.g. “third”, “4^th^”); and/or any reference to a year “(e.g. “2016”, “17”). In addition, we excluded all entries with missing DOIs that could not be found using either manual article title search for journal webpage or Crossref lookup [9]. After excluding these studies, we took a stratified random sample from all four years (i.e. 2014 to 2017) using a randomization sequence. After selecting the sampled studies, we retained only the DOI and study title listed from WoS as unique identifiers.

### Data extraction

A summary of all the data collection with respective sources is found in **Table 2**. Two authors independently reviewed the full-text of publications retrieved from the journal web page to determine eligibility for data collection, wherein any disagreements were resolved by consensus. Following the original WoS search, each study in our sample was individually screened to confirm CIHR funding, as indicated in the acknowledgement section or elsewhere in the paper. Studies were excluded for any of the following: (1) no stated CIHR funding; (2) CIHR funding was provided, but only as part of a graduate or doctoral fellowship (these are exempt from the OA policy) [6]; (3) the full text from the assigned DOI did not match identifying information from WoS, including author names, article title and year of publication in the citation; and/or (4) the paper was a conference abstract or proceeding.

**Table 2:** Summary of all data collected with corresponding sources and databases.

Type of data extraction	Characteristic	Data source/ database
Independent data extraction by two reviewers	CIHR funding status	Full text from journal website
	OA status	Journal website; Google scholar search; Unpaywall
	Year of publication	Journal website
Single reviewer extraction with 10% random check by second reviewer	Corresponding author country	Full text from journal website
	Journal field	Ulrich’s Periodicals Directory
	Journal peer reviewed status	Ulrich’s Periodicals Directory
	Journal country	Ulrich’s Periodicals Directory
	Article citation count (year of publication; total to date)	Web of Science
Single reviewer extraction	Attention Score	Altmetric Explorer

*CIHR*, Canadian Institutes of Health Research; *OA*, open access.

Two authors also screened articles’ OA status using a combination of both manual and automated, using a three step approach that was adapted from prior work [5]. First, both authors screened for OA status by inputting DOIs from each article into the Simple Query Tool from Unpaywall, which is a publicly-accessible platform that automatically indexes papers with open-access status [10]. Second, given the moderate sensitivity of 77% for Unpaywall in detecting OA status [5], two authors manually cross-referenced OA status using the journal webpage linked to the DOI to determine whether an OA article could be retrieved without institutional access. Finally, the same two authors also searched Google Scholar to find all any possible OA versions, excluding versions that were only accessible on academic social networks (e.g. Academia.edu, ResearchGate) or alternative access sites (e.g. SciHub, LibGen), in keeping with other literature [5]. Any study that was accessible using any of the above steps was considered OA and those that were not were considered closed access.

One author extracted the bibliometric data using both the full text retrieved from the journal websites and from relevant databases. Specifically, we collected information on article citation count, Altmetric Attention Score, and journal bibliographic data. For article level citation counts, we used our institutional access to the Web of Science [11]. For the Altmetric Attention Score, we used the Altmetric Explorer, which tracks mentions of the publications across online sites and social media [12]. Journal bibliographic data was retrieved from Ulrich’s Periodicals Directory, a database that provides information on academic publications [12]. Ulrich’s database was used to collect data on journal field, journal country and peer-review status, wherein any categorizations (e.g. journal field as “Medical Sciences”) was taken directly from the database. Another author randomly checked 10% of the secondary data for discrepancies, which were subsequently resolved by consensus. Relevant data for each study are reported in **S1 and S2 Datasets**.

### Outcome measures

The primary outcome measure was whether there was a difference in the proportion of CIHR-funded studies published in 2014 (i.e. before the OA mandate policy change) compared to 2017 (i.e. after the OA mandate policy change). Secondary analyses evaluated the effect of considering the four year period of 2014 to 2017 on the OA status and the impact of OA status on article citation count and attention score.

### Sample size calculation

A power analysis to determine the number of studies for 2014 and 2017 required in the primary analysis of CIHR compliance for OA was computed using G*Power version 3.1.9 [13]. Using a previous study that evaluated OA compliance among international funding agencies, approximately 60% and 40% of CIHR-funded studies were published as OA in 2014 and 2017, respectively [4]. For a two-tailed Fisher’s exact test with proportions of 0.6 and 0.4, an α of 0.05 and a power of 0.9, a total sample size of 282 studies (141 studies per year) was required. To account for the exclusion of studies from the full text review, we decided to screen a total of 1000 studies.

### Analysis

All analyses were conducted in SPSS version 25 (Armonk, NY). Quantitative and categorical variables were presented as means with standard deviation (or median with interquartile range [IQR]) and as count with percentages, respectively. We determined inter-rater reliability for both CIHR funding status (i.e. whether each study was eligible for inclusion in the analysis) and OA status (i.e. whether each study was actually OA or not) for the two raters using Cohen’s kappa coefficient (κ).

To determine whether there was a difference in OA status with CIHR-funded studies published in 2014 and 2017, we used both Fisher exact tests with relative risk (RR) for individual comparisons. We repeated this analysis for CIHR-funded studies published in both 2014 and 2015, compared to studies published in both 2016 and 2017. To determine the effect of our manual approaches in determining CIHR funding and OA status, we conducted the following series of sensitivity analyses using the RR: inclusion of all 1000 randomly selected studies from the systematic search, wherein OA status was manually detected by two reviewers; inclusion of 851 studies screened for accurate CIHR funding, wherein OA status was automatically determined (i.e. using Unpaywall); and inclusion of all 1000 randomly selected studies from the systematic search, wherein OA status automatically detected (i.e. using Unpaywall).

To determine the effect of OA status on article Attention Score and on total citation count, we used Mann-Whitney U tests using OA status Effect size was reported using RR with 95% confidence interval (95% CI) for Fisher exact tests. All statistical tests were two-tailed and considered significant at P<0.05.

## Results

Our search for CIHR funded studies identified an initial 36,144 records over the period of 2014 to 2017. There were 34,920 records after applying our exclusion criteria, of which we randomly selected 250 from each of the four years for a total of 1000 studies. After excluding 149 studies based on our criteria on CIHR funding and publication type, our final sample included a total of 851 records for analysis. The inter-rater reliability was excellent for both CIHR funding status (κ = 0.91) and OA status (κ = 0.92).

A summary of the study characteristics is provided in **Table 3**.

**Table 3:** Study characteristics of included studies with funding from the Canadian Institutes of Health Research (CIHR), *n* = 851.

Characteristic, *n* (%)	
**Year published**	
2014	226 (26.4%)
2015	214 (25.1%)
2016	203 (23.9%)
2017	209 (24.6%)
**Open access**	
Yes	643 (75.6%)
No	208 (24.4%)
**Article type**	
Primary publication	706 (83.0%)
Review/ CPG	133 (15.6%)
Editorial/ Comment	12 (1.4%)
**Corresponding Author Country (Institution)**	
Canada	732 (86.0%)
USA	46 (5.4%)
United Kingdom	14 (1.6%)
Australia	13 (1.5%)
Germany	10 (1.2%)
China	6 (0.7%)
France	6 (0.7%)
Japan	3 (0.3%)
South Korea	3 (0.3%)
Saudi Arabia	2 (0.2%)
Netherlands	2 (0.2%)
Sweden	2 (0.2%)
Spain	2 (0.2%)
Denmark	1 (0.1%)
India	1 (0.1%)
Ireland	1 (0.1%)
Portugal	1 (0.1%)
Scotland	1 (0.1%)
South Africa	1 (0.1%)
Switzerland	1 (0.1%)
Tanzania	1 (0.1%)
Austria	1 (0.1%)
Belgium	1 (0.1%)
**Journal Field**	
Medical Sciences	493 (57.9%)
Biology	192 (22.6%)
Sciences: Comprehensive Works	31 (3.6%)
Chemistry	24 (2.8%)
Public Health	16 (1.9%)
Psychology	14 (1.6%)
Pharmacy	12 (1.4%)
Environmental Studies	9 (1.1%)
Nutrition & Dietetics	7 (0.8%)
Physics	4 (0.5%)
Mathematics	4 (0.5%)
Gerontology & Geriatrics	4 (0.5%)
Drug Abuse & Alcoholism	4 (0.5%)
Children & Youth	4 (0.5%)
Other	33 (3.9%)
**Peer-Reviewed Journal**	
Yes	851 (100.0%)
No	0 (0.0%)
**Journal Country**	
United States	399 (46.9%)
United Kingdom	273 (32.1%)
Netherlands	63 (7.4%)
Switzerland	34 (4.0%)
Canada	26 (3.1%)
Germany	26 (3.1%)
Ireland	14 (1.6%)
India	3 (0.3%)
Australia	2 (0.2%)
Japan	2 (0.2%)
New Zealand	2 (0.2%)
United Arab Emirates	2 (0.2%)
Austria	1 (0.1%)
France	1 (0.1%)
Israel	1 (0.1%)
Italy	1 (0.1%)
Spain	1 (0.1%)

The majority of studies (n = 643; 75.6%) were published open access across the four years included. Most studies were primary publications (n = 706; 83.0%). Canada was the most common country associated with the corresponding author, as determined by the corresponding authors’ primary institutional affiliation (n = 732; 86.0%). All studies were published in peer-reviewed journals and covered a range of different journal fields, wherein medical sciences (n = 493; 57.9%) and biology (n = 192; 22.6%) were the most commonly represented fields.

### Compliance to CIHR open access mandate

Primary outcome data for OA compliance, including sensitivity analyses, is summarized in **Table 4.** In our primary comparison using data from two years that relied on combination OA status determination using both manual and automated approaches (i.e. Unpaywall), we found that CIHR funded studies published in 2017 were significantly less likely to be published as OA compared to those published in 2014 (70.3% vs. 79.6%, respectively; RR = 0.88, 95% CI: 0.79–0.99, P = 0.028).

**Table 4:** Open access compliance among CIHR funded studies across the four year period of 2014 to 2017.

Comparison, *n* (%) ^a^		Open access status	Two year analysis (2014 and 2017)				Four year analysis (2014/2015 and 2016/2017)			
			Published in 2014	Published in 2017	RR (95% CI)	*P* value	Published in 2014 and 2015	Published in 2016 and 2017	RR (95% CI)	*P* value
			*n* (%) ^b^	*n* (%)^b^			*n* (%) ^b^	*n* (%) ^b^		
Primary analysis	Both OA and CIHR status manually determined, 851 (85.1%)	Open	179 (79.6%)	147 (70.3%)	0.88 (0.79–0.99)	0.028*	336 (76.5%)	307 (74.5%)	0.97 (0.90–1.05)	0.493
		Closed	46 (20.4%)	62 (29.7%)	NA	NA	103 (23.5%)	105 (25.5%)	NA	NA
Sensitivity analyses	CIHR funding status not manually checked; manual OA detection, 1000 (100%)	Open	203 (81.2%)	179 (71.6%)	0.88 (0.79–0.97)	0.012*	387 (77.4%)	371 (74.2%)	0.96 (0.89–1.03)	0.238
		Closed	47 (18.8%)	71 (28.4%)	NA	NA	113 (22.6%)	129 (25.8%)	NA	NA
	CIHR funding status manually checked; OA status automatically determined (i.e. Unpaywall), 851 (85.1%)	Open	160 (71.1%)	135 (64.6%)	0.91 (0.80–1.03)	0.149	290 (66.0%)	284 (68.9%)	1.04 (0.95–1.15)	0.371
		Closed	65 (28.9%)	74 (35.4%)	NA	NA	149 (34.0%)	128 (31.1%)	NA	NA
	CIHR funding status not manually checked; OA status automatically determined (i.e. Unpaywall), 1000 (100%)	Open	180 (72.0%)	167 (66.8%)	0.93 (0.83–1.04)	0.208	332 (66.4%)	349 (69.8%)	1.05 (0.97–1.14)	0.249
		Closed	70 (28.0%)	83 (33.2%)	NA	NA	168 (33.6%)	151 (30.2%)	NA	NA

*CIHR*, Canadian Institutes of Health Research.

*All P-values considered significant at P<0.05.

Among the sensitivity analyses for 2014 and 2017 data, we found that manually checking for OA status had an effect on the outcome. Specifically, the comparison of OA status between 2014 and 2017 was not statistically significant in the following situations: when CIHR funding status was manually checked with automatic detection of OA status; and when CIHR funding status was not manually checked with automatic detection of OA status. These discrepancies suggest that there is a non-differential misclassification bias that is driving the RR towards the null, as CIHR funding status misclassification appears to be randomly distributed among OA and non-OA publications. Conversely, another sensitivity analysis found that there was a significant effect of OA status between 2014 and 2017 when CIHR funding status was not manually and OA status was determined using the combination method of manual and automated detection.

In the secondary comparison using data from all four years, we found that there was no significant difference in the proportion of CIHR funded studies published as OA in both 2017 and 2016 compared to both 2015 and 2014 (74.5% vs. 76.5%, respectively; RR = 0.97 95% CI: 0.90–1.05, P = 0.493). Sensitivity analyses among this group did not identify any discrepant trends.

### Citation count and attention score

Secondary outcome data for the effect of OA status on article citation count and Attention score is summarized in **Table 5.** There was a significant difference for citation count from the year of publication (U = 61546.5, P = 0.020), total citation count (U = 50959.5, P<0.001), and attention score (U = 37088.0, P<0.005), wherein OA publications had higher counts on all three measures.

**Table 5:** Altmetric Attention Score and citation count among CIHR funded studies across the four year period of 2014 to 2017.

	Open access, median (IQR)	Closed access, median (IQR)	*P* value
**Altmetric Attention Score**	3.0 (10.0)	2.0 (7.0)	0.020*
**Citation count (within year of publication)**	0 (2.0)	0 (1.0)	<0.001*
**Citation count (total)**	17.0 (25.0)	9.0 (15.0)	<0.005*

*CIHR*, Canadian Institutes of Health Research; *IQR*, interquartile range.

*P <0.05.

## Discussion

In our analysis of 851 studies receiving CIHR funding that were required to publish their findings as OA, we found that there was a significant decrease in the number of studies that actually complied with the CIHR OA mandate. Specifically, the percentage of CIHR funded studies published as OA significantly decreased from 79.6% in 2014 to 70.3% in 2017. When considering all four years of 2014 to 2017, the trend of decreasing OA compliance remained, though the difference in the OA proportion of studies from 2014 and 2015 compared to 2016 and 2017 was not statistically significant. Finally, we found that there was a higher citation count and higher Altmetric score among CIHR funded studies published as OA compared to those as closed access across all four years.

Our findings provide a robust confirmation of existing literature on OA compliance among CIHR-funded research. In particular, our approach, which used a combination of manual and automated methods, substantiated the previous claim that there was a decrease of approximately 10% in OA compliance among CIHR funded studies [4]. We do note, however, that our estimate for the percentage of OA studies is discrepant with the previous study on the topic, as it reports that approximately 60% and 40% of CIHR funded studies were published as OA in 2014 and 2017, respectively, which contrasts with our own estimates of 79.6% and 70.3% for those respective years. These discrepancies may be explained, at least partially, by the previous study’s sole use of an automated method for OA detection (i.e. Unpaywall), which is reported to have moderate sensitivity [5]. In addition, it is possible that there was a retroactive increase in OA publication since 2018 (i.e. the time of the Larivière et al study) and 2020 (i.e. the time of the current analysis), as both authors and journals may have made previously closed access articles OA, such as through digital archiving.

We note the strengths of this study. The primary strength is the focused nature of our investigation, as our sample size allowed for an in-depth exploration and verification that is unlikely to be feasible with a large scale analysis. Specifically, our sample of 1000 allowed for independent verification of both CIHR funding status and OA status using a combination of manual and automated methods. Additionally, we employed a series of comprehensive sensitivity analyses that accounted for the effect of our manual efforts to determine both the presence of CIHR funding and OA status.

There are several limitations of this study. First, our reliance on author self-reporting of study funding may have introduced bias into the included studies, as some studies may not have indicated CIHR funding. Second, though we were careful to exclude any studies exempt from the OA mandate based on award type (e.g. graduate and/or post-doctoral CIHR funding), it is possible to have missed studies in which award type was not indicated. Third, we could not discriminate between the different types of OA as either primarily a journal or repository, which restricted our inferences in this regard. Fourth, though we employed a sample size calculation to determine the number of required studies, it is nonetheless possible sampling error may have occurred, which would affect the representativeness of our findings. Finally, we did not *a priori* pre-register our protocol, which may have introduced bias into the analysis of our findings.

With these limitations in mind, we highlight several implications of our findings. Specifically, we posit that the significant decrease in the amount of CIHR funded studies published as OA since 2015 was due to, at least partially, the CIHR OA policy change. This assertion is put forward in the large scale study on the topic [4]. Conversely, one alternative hypothesis is that there is a decline in OA compliance unrelated to the policy change, which is a possibility that cannot be definitively ruled out. Nevertheless, that the decline is occurring remains a cause for concern. One counterpoint to our finding that the approximate 10% drop of OA publication from 2014 to 2017, while statistically significant, may not be “clinically” significant–that is, this decline may be within tolerable limits. The natural response to this point is to contextualize the trend within the previous finding that Canadian public funding agencies (including CIHR) have among the lowest levels of OA compliance worldwide [4]. For comparison, the National Institute of Health (NIH), the major American federal research funding agency, has more than 85% of its funded research published as OA. Therefore, any decline, represents an exacerbation of an already poor condition. Furthermore, the consequences of poor OA compliance among CIHR studies are two-fold: first, authors are potentially missing out on practical benefits to OA publication, such as increased citation and visibility [14]; and second, and perhaps more importantly, the public is being deprived access to research funded by taxpayers [3].

Future research should aim primarily to confirm these findings, ideally with a larger dataset. In addition, future studies should investigate the effectiveness of strategies implemented by other public funding agencies that have demonstrably high OA compliance in order to determine whether it effectively improve OA compliance among CIHR funded research. Using the NIH as an exemplar, which has among the highest levels of OA compliance according to one study [4], we strongly suggest exploration of three interventions:. first, a database linking awarded grants with their associated publications can allow for prospective tracking of OA compliance, such as the Research Portfolio Online Reporting Tools (RePORT) at NIH [15]; second, reinstatement of the policy for immediate deposition of CIHR funded studies into OA repositories, instead of 12 months after publication; and finally, implementation of sanctions, such as withholding of funding from authors who do not publish their works as OA, an intervention used by the NIH [16].

## Conclusions

In summary, we found that there was a significant decrease in the proportion of CIHR funded studies published as OA from 2014 compared to 2017, though this difference did not persist when comparing both 2014–2015 to 2016–2017. Even accounting for this discrepancy, there appears to be a discernible decline in the proportion of OA studies supported by CIHR funding. One explanation for this decline may be attributable to CIHR’s OA policy change in 2015. Future research should aim to develop and evaluate interventions that can improve OA compliance, such as the automated linkage of CIHR grants to published studies.

## Supporting information

S1 Appendix Meta-epidemiologic reporting checklist. (DOCX)

S1 File Search strategy. (DOCX)

S1 Dataset Primary outcome data for all included studies. (XLSX)

S2 Dataset Secondary outcome data for all included studies. (XLSX)
