# Supplementary material for: Do authors of research funded by the Canadian Institutes of Health Research comply with its open access mandate?: A meta-epidemiologic study
Source: PLoS One. 2021 Aug 24;16(8):e0256577. doi: 10.1371/journal.pone.0256577 (PMC8384194; doi:10.1371/journal.pone.0256577)
Supplement: S1 File — (DOCX) [file pone.0256577.s002.docx]

**S1 File**

**Supplement 1: Comprehensive Search Strategy**

Web of Science

FO=(CIHR) AND FO=(Canad* Institute* of Health Research) OR FO=(Canad* Institute* for Health Research) OR FT=(Canad* Institute* AND Health research) OR FT=(New Frontiers in Research Fund) OR FT=(Vanier Canada Graduate Scholarships) OR FT=(Banting Postdoctoral Fellowships) OR FT=(Antimicrobial Resistance) OR FT=(AMR) OR FT=(Canadian Epigenetics, Environment, and Health Research Consortium) OR FT=(CEEHRC) OR FT=(Canadian Longitudinal Study on Aging) OR FT=(CLSA) OR FT=(Canadian Research Data Centre Network) OR FT=(CRDCN) OR FT=(Canadian Research Initiative in Substance Misuse) OR FT=(CRISM) OR FT=(CIHR Dementia Research Strategy) OR FT=(Collaborative Health Research Projects) OR FT=(Community-Based Primary Health Care) OR FT=(CBPHC) OR FT=(Drug Safety and Effectiveness Network) OR FT=(DSEN) OR FT=(eHealth Innovations) OR FT=(Environments and Health) OR FT=(Evidence-Informed Health Care Renewal) OR FT=(EIHR) OR FT=(Health Research Rapid Response) OR FT=(Health and Productive Work) OR FT=(Health Life Trajectories Initiative) OR FT=(HeLTI) OR FT=(Indigenous Health Life Trajectories Initiatives) OR FT=(I-HeLTI) OR FT=(Hepatitis C Research Initiative) OR FT=(HIV/AIDS Research Initiative) OR FT=(Inflammation in Chronic Disease) OR FT=(Integrated Cannabis Research Strategy) OR FT=(Network Environments for Indigenous Health Research) OR FT=(NEIHR) OR FT=(Pandemic Preparedness Strategic Initiative) OR FT=(PPSRI) OR FT=(Pathways to Health Equity for Aboriginal Peoples) OR FT=(Personalized Health) OR FT=(Personalized Medicine) OR FT=(Research in Substance Use) OR FT=(Strategy for Patient-Oriented Research) OR FT=(SPOR) OR FT=(Transitions in Care) OR FT=([CIHR Gold Leaf Prize for Impact](https://www.researchnet-recherchenet.ca/rnr16/vwOpprtntyDtls.do?prog=2884&view=currentOpps&org=CIHR&type=EXACT&resultCount=25&sort=program&next=1&all=1&masterList=true)) OR FT=([CIHR Gold Leaf Prize for Discovery](https://www.researchnet-recherchenet.ca/rnr16/vwOpprtntyDtls.do?prog=2882&view=currentOpps&org=CIHR&type=EXACT&resultCount=25&sort=program&next=1&all=1&masterList=true)) OR FT=([CIHR Gold Leaf Prize for Outstanding Achievements by an Early Career Investigator](https://www.researchnet-recherchenet.ca/rnr16/vwOpprtntyDtls.do?prog=2883&view=currentOpps&org=CIHR&type=EXACT&resultCount=25&sort=program&next=1&all=1&masterList=true)) OR FT=([CIHR Gold Leaf Prize for Transformation](https://www.researchnet-recherchenet.ca/rnr16/vwOpprtntyDtls.do?prog=2881&view=currentOpps&org=CIHR&type=EXACT&resultCount=25&sort=program&next=1&all=1&masterList=true))
